# Navigating the expanded access investigational new drug protocol for tecovirimat: lessons learned from a public–private hospital partnership during the 2022 NYC mpox outbreak

**DOI:** 10.1017/ash.2023.194

**Published:** 2023-06-29

**Authors:** Ofole Mgbako, Justin Chan, Robert A. Pitts, Madeline A. DiLorenzo, Dorothy Knutsen, Dana Mazo

**Affiliations:** 1 Division of Infectious Diseases and Immunology, Department of Medicine, NYU Langone Health, New York, NY, USA; 2 Division of Infectious Diseases, Department of Medicine, New York City Health + Hospitals/Bellevue, New York, NY, USA

## Abstract

During the 2022 mpox outbreak, tecovirimat was accessed through an expanded access investigational new drug (EA-IND) protocol. We leveraged a unique public/private hospital partnership in New York City to create a novel infrastructure to navigate the EA-IND’s regulatory requirements and rapidly provide tecovirimat to patients.

## Introduction

The recent global mpox outbreak began in the United States on May 19, 2022, with New York City (NYC) accounting for 15%–20% of new infections at its peak.^1,2^ Patients presented with severe morbidity, from ocular involvement to painful anorectal lesions and proctitis. Shortly after the outbreak began, the US Centers for Disease Control and Prevention (CDC) established an expanded access investigational new drug (EA-IND) protocol for tecovirimat use in non-variola orthopoxviruses including mpox.^3^ With cases rising rapidly in NYC, NYC Health + Hospitals/Bellevue (Bellevue), the main referral hospital for NYC’s municipal health system, and New York University Langone Health (NYULH), an academic medical center with four acute care hospitals in NYC and Long Island, established a joint mpox treatment model with assistance from the NYC Department of Health and Mental Hygiene (NYC DOHMH). Here, we discuss how we: (1) created infrastructure to reach the most affected populations and rapidly scale up treatment capacity and (2) navigated ongoing regulatory requirements associated with obtaining antivirals via the EA-IND protocol.

## The EA-IND process: Leveraging the Bellevue and NYULH partnership during a public health emergency

**Figure 1.**
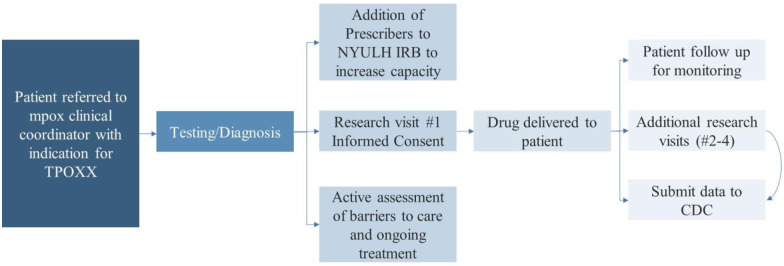
Steps involved in obtaining and delivering tecovirimat at Bellevue and NYULH.

Many regulatory requirements were involved in obtaining tecovirimat through an EA-IND protocol. The initial EA-IND protocol required written informed consent, four clinical visits, and multiple clinical reports completed by prescribers and submitted to the CDC (Figure 1). While the EA-IND did not technically involve research, it involved clinical care using a non-FDA-approved treatment, thus requiring IRB oversight. The EA-IND allowed use of the CDC IRB, yet internal policies at many institutions, including ours, required review from the local institutional IRB. At the start of the outbreak, the CDC did not provide reliance agreements to institutional IRBs to approve use by an external central IRB. Despite the EA-IND involving solely clinical care, local IRBs frequently required following the processes they have in place for clinical research including completion of time-consuming clinical research training by all staff working with patients and submission of protocol modifications for each new prescriber.

Given these regulatory challenges, infectious disease physicians at Bellevue and NYULH had to create a new infrastructure for rapid treatment. Infectious diseases clinicians at both institutions are all faculty at NYU Grossman School of Medicine, collaborate regularly on research and education initiatives, and are overseen by the NYULH IRB. Both institutions also have infrastructure dedicated to preparing for novel infectious diseases outbreaks. Bellevue is one of 13 federally funded Regional Emerging Special Pathogens Treatment Centers and NYULH has a standing High Consequence Pathogens Committee, which is an interdisciplinary group that prepares the hospital system for an influx of patients due to an emerging communicable disease.^4^

**Figure 2.**
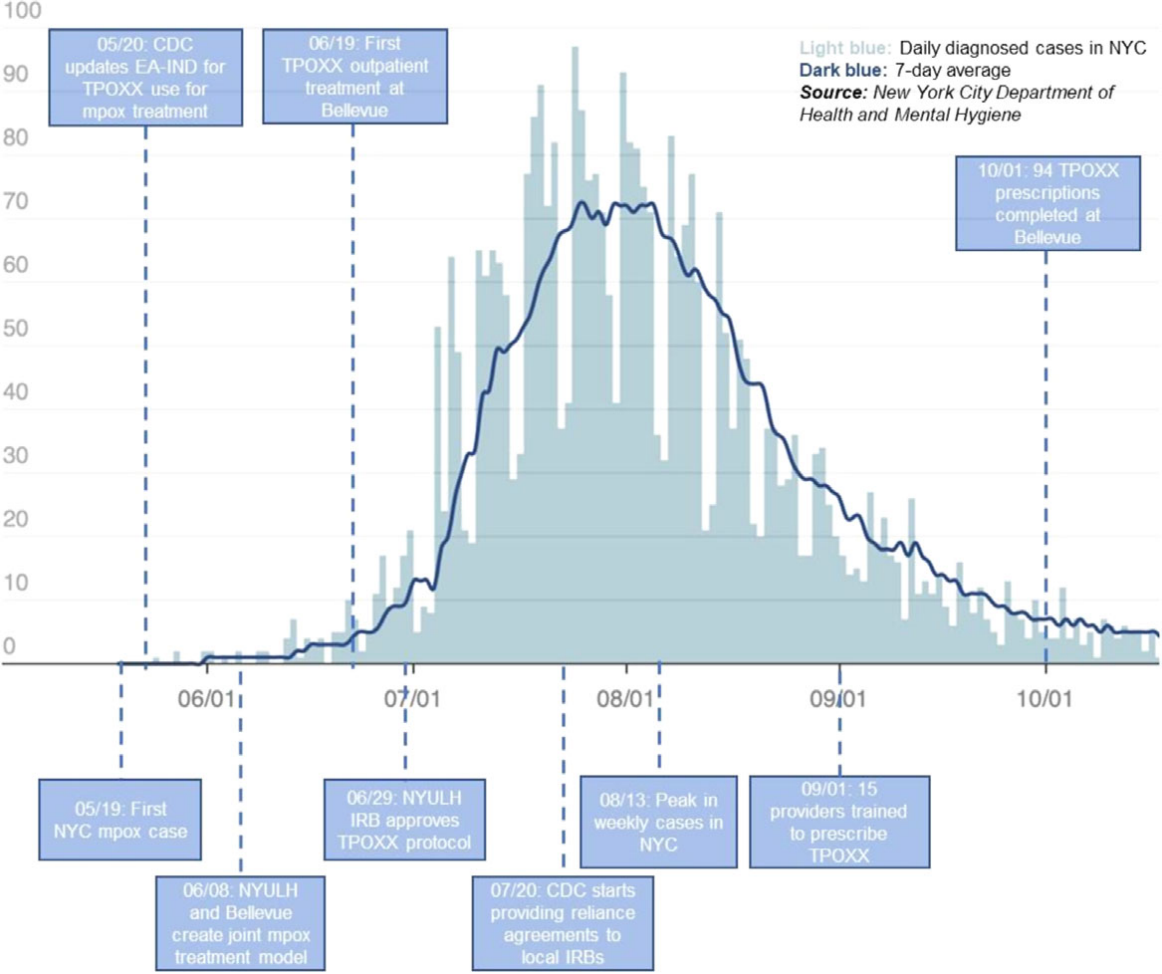
Epidemiological curve of mpox outbreak in NYC and timeline of the tecovirimat (TPOXX) roll-out at NYULH and Bellevue.

NYULH provided the first dose of tecovirimat in NYC on June 8, 2022. Preparing for the coming wave of new infections, we then convened infectious diseases faculty and infection prevention and control leadership early at our institutions to address barriers for providing treatment. First, we focused on approval from the NYULH IRB by directly engaging with IRB leadership on the unique circumstances of needing local IRB approval for treatment during a public health emergency. While our NYULH IRB application was approved relatively quickly, the processes required to add prescribers meant we initially only had one physician at each site prescribing tecovirimat to patients via outpatient telehealth visits, even as the number of mpox patients requiring treatment rose dramatically (Figure 2).^5^ As we needed to rapidly expand our cadre of tecovirimat prescribers to meet increased demand, our IRB assigned us a specific staff member whom we could alert multiple times a day to approve new personnel modifications quickly. Unfortunately, the IRB requirement for clinical research training was onerous since many potential tecovirimat providers did not participate in research so they had to complete multiple e-modules.

Additionally, while some institutions successfully leveraged existing resources, often Ryan White HIV clinics or AIDS Clinical Trial Group (ACTG) sites given the high rate of HIV-mpox co-infection, neither NYULH nor Bellevue has an ACTG site^6,7^ However, we trained a group of 15 infectious disease and internal medicine faculty who could see patients during evening hours for moonlighting reimbursement. Starting in August, the NYU Langone Vaccine Center provided administrative support on a volunteer basis. In addition, Bellevue hospital leadership hired three short-term nurse practitioners to expand capacity for treatment and NYULH hospital leadership approved short-term funding for an mpox clinical coordinator, who was an infectious disease faculty member familiar with the clinical care delivery processes at Bellevue and NYULH. These measures increased our ability to treat patients with mpox in a timely manner and rapidly scale-up services, and Bellevue and NYULH provided a large proportion of the earliest antiviral treatments for mpox in NYC.^8^

## Navigating barriers to equitable care

There were many barriers to equitable access to care during the mpox public health emergency. The requirement for IRB oversight led to extra paperwork for non-English-speaking patients and members of vulnerable populations, such as children, pregnant women, incarcerated individuals and those with limited capacity for consent. In the interest of submitting the protocol quickly in the setting of a public health emergency, the initial version did not include these vulnerable groups. Existing IRB regulations are meant to protect these groups from potential exploitation in research, yet they inadvertently perpetuated inequities in care.

For example, both Bellevue and NYULH serve large Spanish-speaking and other immigrant populations and Bellevue provides inpatient care and outpatient specialty services to all male patients incarcerated in the NYC jail system. We worked with our IRB to ensure inclusion of non-English-speaking groups by pairing patient-provider dyads who spoke the same language when possible and using language interpreter services to translate consents, short forms, treatment plans, and discharge instructions into the patient’s preferred language. While our submission of necessary IRB modifications for incarcerated individuals and other vulnerable groups was pending, we worked closely with the IRB on clinical cases that may have required tecovirimat for patients who were part of a population not included in the initial submission, in case emergency exceptions needed to be made.

Of note, many of our patients with mpox were uninsured or under-insured, which raised patients’ concerns about the potential cost of the multiple treatment visits required by the EA-IND protocol. We actively partnered with our financial counseling office to ensure all patients with mpox-related appointments were screened for insurance eligibility prior to their first appointment and that ineligible patients were fee-coded based on their existing income. This minimized out-of-pocket expenses for mpox testing and treatment as much as possible.

## Lessons learned

The mpox outbreak in NYC presented many challenges for Bellevue and NYULH, including an initial lack of treatment infrastructure, IRB restrictions, limited staff, and lack of administrative support to adhere to CDC protocols for tecovirimat access. Infectious disease faculty physicians at NYULH and Bellevue collaborated effectively by leveraging public/private infectious diseases partnerships across our health systems.

Our experience has revealed crucial lessons that can be applied to the next potential infectious disease outbreak if regulatory barriers to treatment access are in place. First, a strong partnership with IRB leadership is needed to streamline local approval processes that are not intended for research. This includes rapid review of protocols, allocation of support staff to ensure IRB-related procedures are completed, and openness to adapt IRB processes as the IRB oversight mechanisms for research differ from that required for clinical care administered under an EA-IND protocol. Second, communication and collaboration with city and/or public health officials are crucial. Our DOHMH colleagues helped familiarize us with the EA-IND protocols, provided feedback to the CDC on the required paperwork, and assisted with management of complicated cases. Third, hospital leadership support is necessary to secure dedicated resources for staffing. Without funding for temporary staff and a dedicated mpox care coordinator, we would not have been successful in establishing an equitable mpox treatment program while avoiding staff burnout.

Finally, there were significant inequities in treatment access for communities most affected by the mpox outbreak, namely racial/ethnic, sexual, and gender minorities. Working with our IRB, we were able to serve our most marginalized populations. We addressed inequities in access to care as they were identified (eg, active insurance evaluation and assistance, addressing language barriers). However, in future infectious disease public health emergencies, there should be an equity-focused preparedness plan in place, applicable to other pathogens involving an EA-IND protocol, that ensures quick access to therapeutics for all affected communities.

## Recommendations for public health authorities on reducing hurdles for hospitals

In the event of a future outbreak involving experimental vaccines or therapeutics, there are important steps that would make it much easier for hospitals to deliver care and work better with public health authorities. First, there should be public health emergency funds set aside for infectious disease outbreaks to cover extra administrative support to address the regulatory burden of accessing treatment. Second, there should be a system in place with local IRBs for emergency processes that balance protection of vulnerable populations with achieving equity for the most marginalized. In addition, specific IRB personnel with knowledge of clinical care delivery systems should be identified. We also recommend unique collaborations between public and private medical institutions, often which have unique infrastructure in place that can support patients rapidly in an infectious diseases outbreak like mpox. For example, private institutions often lack the ability to expand treatment to populations with a lot of social needs and support, while public institutions often lack the infrastructure to deal with regulatory burdens of IRB processes. New public–private partnerships can make up for healthcare system deficiencies within each model during an emergency.

**Figure 3.**
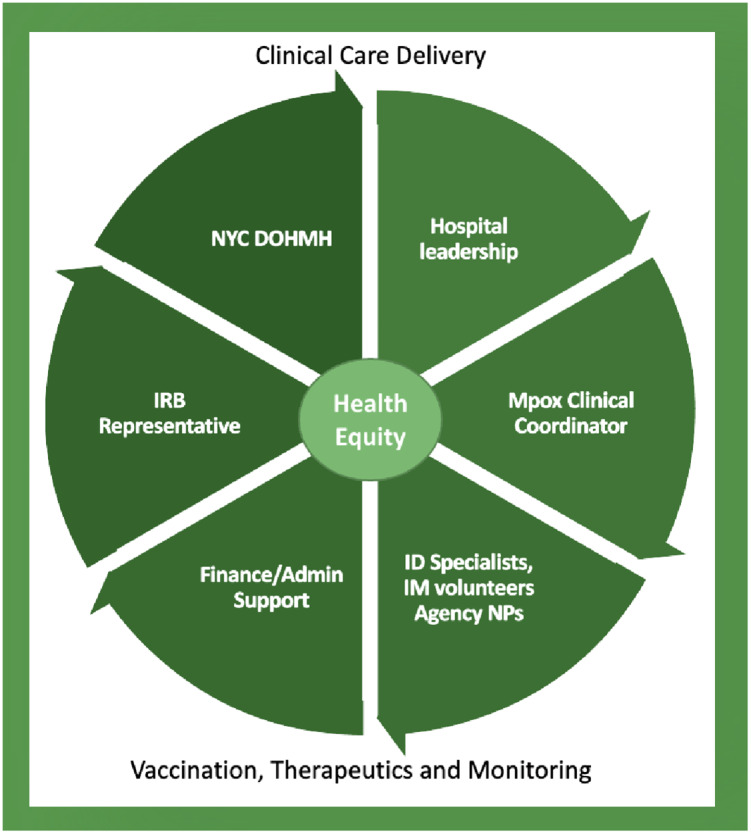
Strike team members at NYULH and Bellevue for mpox outbreak focused on clinical care delivery, vaccinations, therapeutics and monitoring, and health equity. ID, infectious diseases; IM, internal medicine; NP, nurse practitioners.

Lastly, we believe that hospitals systems should proactively assemble a “strike team” to be called upon in the setting of a new outbreak. The team should focus on establishing clinical care delivery workflows, initiating a model for vaccination, therapeutics and monitoring, and centering an approach to achieve health equity for the most vulnerable populations in the midst of an emergency. Our strike team started with infectious diseases specialists engaging financial services/administrative support and IRB staff, as well as NYC DOHMH and hospital leadership to stand up a clinical care delivery model (Figure 3). Then, an mpox clinical coordinator across both systems was appointed and oversaw infectious diseases clinicians, and trained internal medicine volunteers and agency nurse practitioners (Figure 3). Public health authorities could support and promote these recommendations by convening practitioner groups to develop best practices and then support hospitals to enact these processes when a public health emergency arises.
